# Novel wildfire regimes under climate change and human activity: patterns, driving mechanisms and ecological impacts

**DOI:** 10.1098/rstb.2023.0446

**Published:** 2025-04-17

**Authors:** Zehao Shen, Kate Giljohann, Zhihua Liu, Juli Pausas, Brendan Rogers

**Affiliations:** ^1^ Institute of Ecology, College of Urban & Environmental Sciences, Peking University, Beijing 100871, People’s Republic of China; ^2^ Commonwealth Scientific and Industrial Research Organisation, Canberra, Victoria, Australia; ^3^ Chinese Academy of Science Institute of Applied Ecology, Shenyang 110016, People’s Republic of China; ^4^ CIDE/CSIC, Moncada, Valencia 46113, Spain; ^5^ Woodwell Climate Research Center, Falmouth, MA, USA

**Keywords:** fire activity, fire-prone vegetation, fire regime change, climate change, human activity, ecological impacts, ecosystem feedback

## Abstract

Fire regime refers to the statistical characteristics of fire events within specific spatio-temporal contexts, shaped by interactions among climatic conditions, vegetation types and natural or anthropogenic ignitions. Under the dual pressures of intensified global climate changes and human activities, fire regimes worldwide are undergoing unprecedented transformations, marked by increasing frequency of large and intense wildfires in some regions, yet declining fire activity in others. These fire regime changes (FRC) may drive responses in ecosystem structure and function across spatio-temporal scales, posing significant challenges to socio-economic adaptation and mitigation capacities. To date, research on the patterns and mechanisms of global FRC has rapidly expanded, with investigations into driving factors revealing complex interactions. This review synthesizes research advancements in FRC by analysing 17 articles from this special issue and 249 additional publications retrieved from the Web of Science. We systematically outline the key characteristics of FRC, geographical hotspots of fire regime transformation, critical fire-prone vegetation types, primary climatic and anthropogenic drivers and ecosystem adaptations and feedbacks. Finally, we highlight research frontiers and identify key approaches to advance this field and emphasize an interdisciplinary perspective in understanding and adapting to FRC.

This article is part of the theme issue ‘Novel fire regimes under climate changes and human influences: impacts, ecosystem responses and feedbacks’.

## 1. Introduction

Recent extreme wildfires in North America [1,2], Australia [3], Mediterranean regions [4] and other regions signal a potential planetary shift into a new era of fire regimes, particularly evident in high-latitude Northern Hemisphere ecosystems [5,6]. Paradoxically, traditionally fire-prone biomes—including sub-Saharan savannas, Brazilian cerrado and Southeast Asian forests—reveal declining fire activity, a trend largely attributable to anthropogenic modifications of land use patterns and fire management practices [7–9]. A succession of high-impact studies (e.g. [2,10]) continue to validate predictions of significant spatio-temporal realignments in global wildfire patterns, a transformation that has amplified international scientific concern [11]. For the first time in Earth’s history, human activities may rival or surpass natural forces as the primary driver of wildfire dynamics at global scales. The anthropogenic influence manifests not only through altered fire regimes but also more fundamentally through the restructuring of spatio-temporal patterns of biodiversity responses and driving feedbacks at species and ecosystem levels [12,13].

Fire regime encompasses the temporal patterns, intensities and spatial extents of fire events within a defined geographical area and time frame, shaped by interactions among climatic conditions, fuel availability and ignition sources [14]. The complex interplay between shifting wildfire regimes and their dual drivers—natural processes and anthropogenic activities—has positioned global environmental change as a catalyst for wildfires, and vice versa, with unprecedented ecological and socio-economic consequences [15]. This phenomenon is driving the worldwide development of strategies and policies for land management, vegetation conservation, fire mitigation and adaptation. To address these challenges, it becomes imperative to systematically analyse emerging wildfire patterns across biomes, while investigating climate–fire–vegetation interactions through multi-scale analyses that account for both natural and human-modified landscapes. This scientific imperative is evidenced by the rapid recent growth in wildfire-related publications and the number of special issues in prominent journals dedicated to advancing fire science (table 1)—a clear testament to the urgency of understanding wildfire dynamics in the Anthropocene.

**Table 1.** The published special issues on fire and fire regime in different journals.

journal name	year	vol.(iss.)	theme
*Global Change Biol*.	2009	15(3)	Future wildfire and climate change [16]
*Phil. Trans. R. Soc. B*	2016	371(1696)	The interaction of fire and mankind [17]
*Int. J. Wildland Fire*	2019	28(5, 7)	Fire regime and ecosystem responses: adaptive forest management in a changing world, part I, II [18,19]
*Environ. Res. Lett*.	2016−2018	11−13	Focus on changing fire regimes: interactions with climate, ecosystems and society [20]
*Plant Ecol*.	2022	223(7)	Impacts of climate change and altered fire regimes on plant populations, species and ecosystems [21]
*Divers. Distrib*.	2022	28(3)	Fire ecology for the 21st century: conserving biodiversity in the age of megafire [22]
*Front Ecol. Evol.*	2022	10	Fire regimes in desert ecosystems: drivers, impacts and changes [23]
*One Earth*	2024	7(6)	The burning challenge: seeking a sustainable path for people and fire [24]

These sustained efforts underscore the urgent need to understand the spatio-temporal variation of fire regimes across scales. This is more meaningful to fill the knowledge gaps in regional and ecosystem coverage beyond existing studies. Our current understanding of fire regime characteristics and their driving mechanisms is challenged by novel trends in both climate and human activities, which have resulted in dramatic alterations in global wildfire patterns over recent decades [7,25]. Therefore, elucidating the feedback mechanisms between fire regimes and ecosystems, or species, holds critical significance for predicting ecological consequences of future fire dynamics.

Given the inherent complexity of cause–effect relationships and the multiscale, multidimensional nature of ecological impacts, an urgent imperative emerges to (i) synthesize multidisciplinary observational data across spatio-temporal scales; (ii) unravel the tripartite interactions between global climate change, fire regime changes (FRC) and ecosystem responses; and (iii) quantify anthropogenic influences across this nexus. Such efforts are critical for predicting and mitigating evolving wildfire risks and associated threats to ecosystem functions—a challenge that constitutes the primary motivation behind the present thematic issue. Through the lens of FRC, this article synthesizes the research literature of the last three decades (1995−2025) and highlights the contribution of this collection. We then review the main features, global hotspots, natural and human drivers and ecological impacts of FRC, while outlining future research trajectories for understanding climate–human–fire interactions and their cascading ecological consequences.

## 2. Literature statistics on fire regime change

To elucidate current understanding of FRC, we conducted a systematic literature review using the Web of Science database (https://webofscience.clarivate.cn/wos/alldb/basic-search). Our search targeted publications on the theme of FRC with the query: Title = (‘*fire regime’ OR ‘*fire activity’) AND (‘shift’ OR ‘change’ OR ‘modif*’ OR ‘alter*’) (Search date: 31 December 2024). After manual screening for relevance, we identified 247 documents (231 research articles, eight reviews, six editorials and two conference proceeding papers) published across 99 peer-reviewed journals, mostly in ecology and environmental sciences. Among them, *Global Change Biology* (11), *International Journal of Wildland Fire* (10) and *Quaternary Science Reviews* (10) published most papers on the topic.

### (a) Temporal and spatial patterns of literature on fire regime changes

Although the publishing of peer-reviewed papers in this field was initiated in 1981, the steady publication of FRC studies occurred only after 1995, and the number of papers began to prominently increase since 2007 (see appendix).

**Figure 1.**
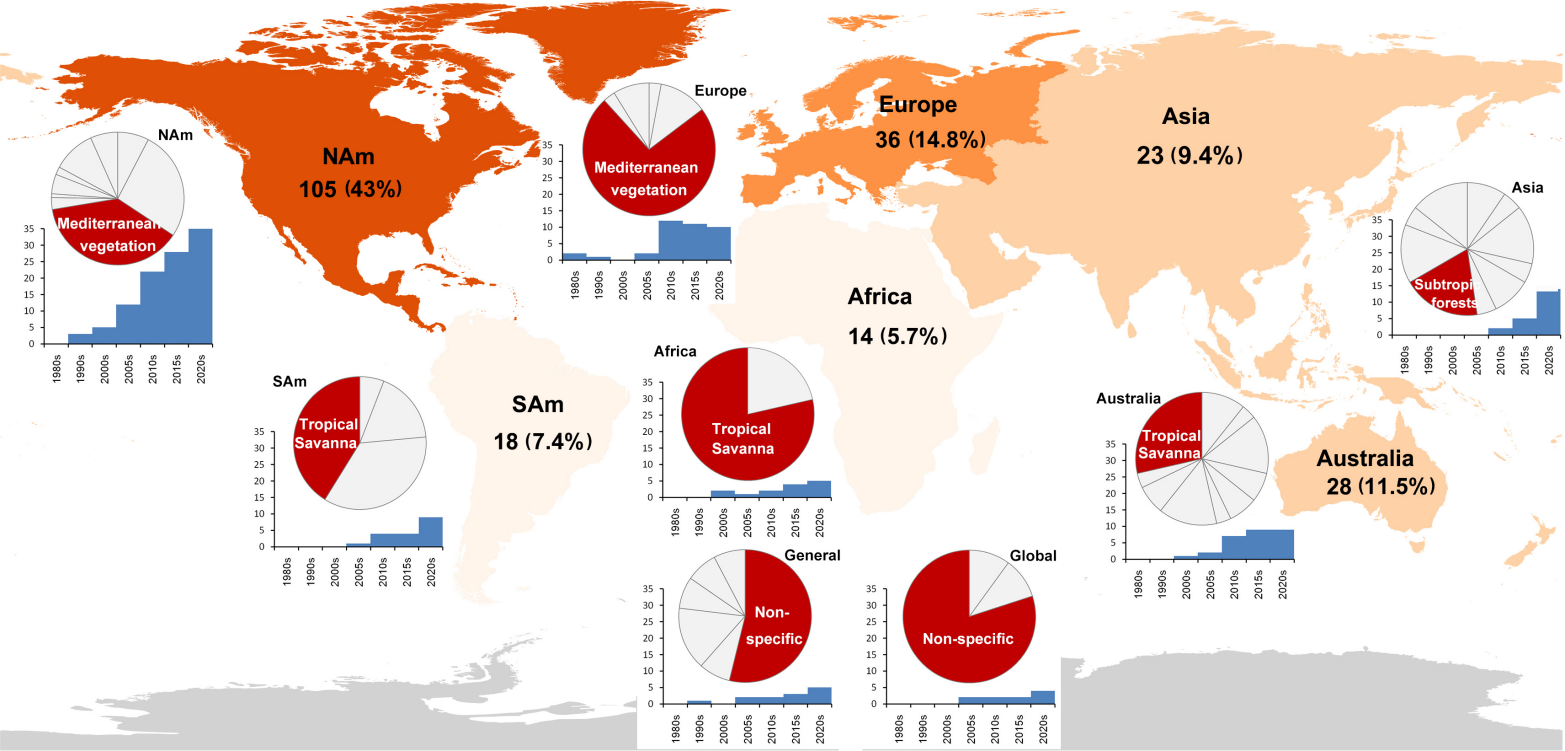
The temporal and geographical distributions of FRC studies. Pie charts depict the number of studies by self-reported biome, and histograms show the number of studies by decade of publication. The red section in each pie represents the most studied ecosystem type in each continent.

The 247 FRC papers exhibit marked temporal and spatial disparities across continents and ecosystems (figure 1). North America dominates the literature (105 studies), with Mediterranean vegetation (primarily in California) as the most studied ecosystem, followed by boreal forests in Canada and the northwestern US. North American studies began in 1995 and expanded rapidly in recent decades. Europe ranks second (36 studies), also emphasizing Mediterranean regions. Despite pioneering work starting in 1981, European research surged post-2005 and plateaued after 2010. Australia contributes 28 studies, emerging in the 2000s and mainly focusing on tropical savannas, Mediterranean shrublands and eucalypt forests. Asian studies started around 2010 and grew swiftly in the past decade (23 studies), primarily addressing subtropical forests, Far Eastern boreal forests and Southeast Asian tropical savannas. South America accounts for 18 studies, initiated in 2005 and centred on tropical savannas, temperate forests and rainforests. Africa, despite long-standing wildfire research, contributes only 14 studies (5.7%), predominantly concerning savannas. Ten global scale studies and 13 general studies (mostly reviews and editorials) analyse FRC across worldwide ecosystems. Notably, Northern Hemisphere research emphasizes extra-tropical vegetation (especially Mediterranean ecosystems), while Southern Hemisphere studies primarily focus on tropical savannas.

### (b) Key topics of fire regime change studies

**Figure 2.**
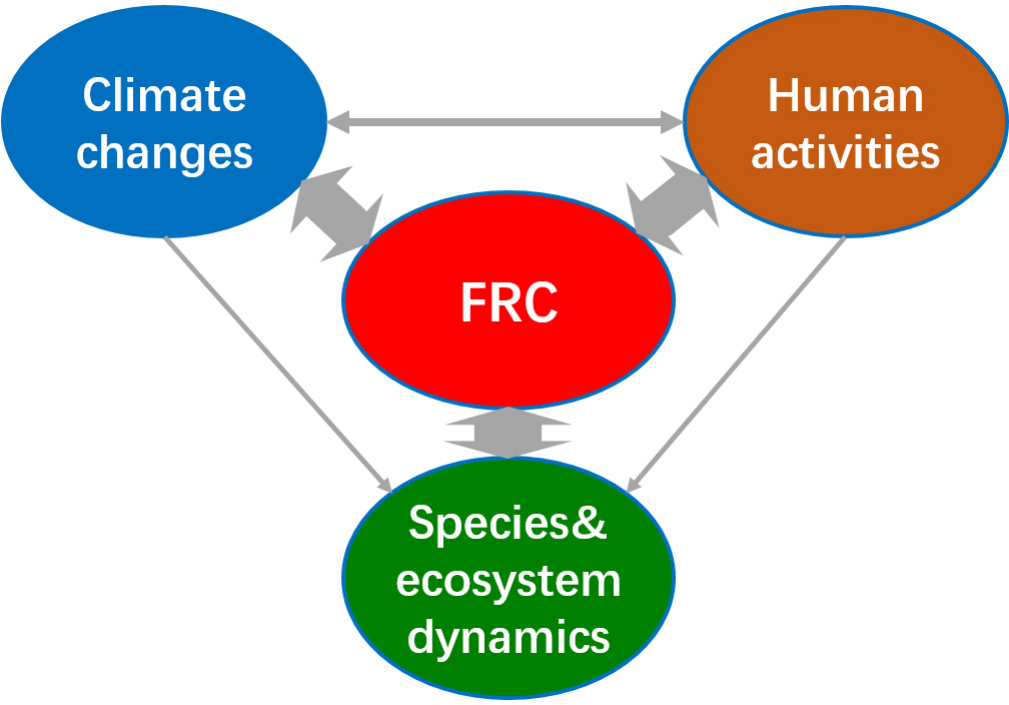
The conceptual framework of fire regime changes (FRC) studies.

FRC studies addressed either the fire regime features (such as fire frequency, size, intensity, seasonality, etc.), their drivers (natural, anthropogenic or both) or their ecological effects (on species, community or ecosystems) (figure 2). An analysis of the literature reveals distinct patterns in research focus: nine studies specifically investigate shifts in fire regime features. A total of 150 studies examine two interconnected dimensions: changes in fire activity alongside either their drivers (e.g. climatic or anthropogenic factors) or their ecological/societal impacts. Of these, 81 studies address three aspects, including fire regime features, natural (e.g. climate variability) and/or anthropogenic drivers (e.g. land use changes, fire suppression policies), and also fire effects. Only seven studies comprehensively link all four dimensions: FRC, natural drivers (primarily climate change), anthropogenic drivers (land use changes, policy interventions) and the cascading effects of fires on ecosystems, environmental systems and human livelihoods.

**Figure 3.**
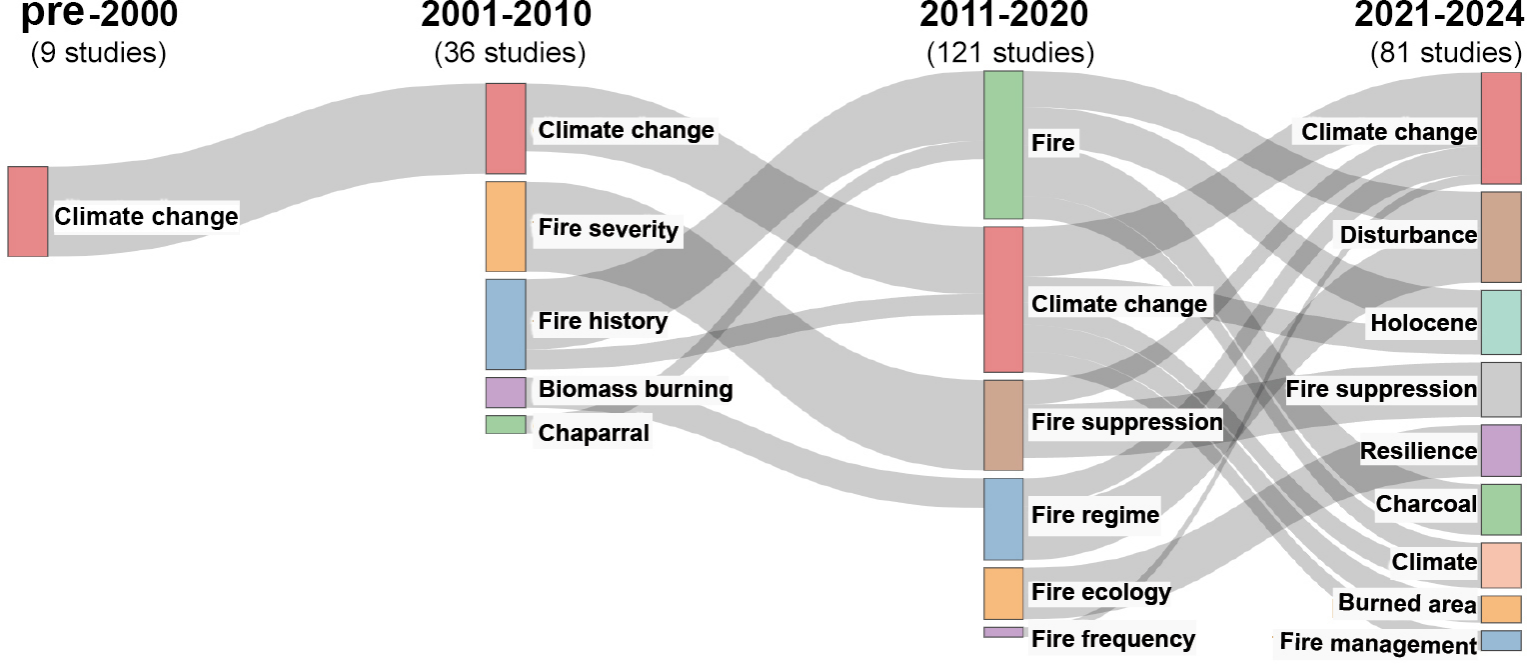
The development of thematic contents in fire regime shift studies.

By tracing shifts in the categorical alignment between cited papers and their citing counterparts, the thematic evolution of FRC research across two consecutive periods is identified based on key words of the papers and reveals distinct trends across the last three decades (figure 3). A connection between studies in neighbouring periods is established based on the theme category of each cited paper and the citing papers. The nine pre-2000 studies primarily focus on the impact of climate change on FRC. The scope of research expanded during 2001−2010, with 36 studies not only continuing to explore climate change but also focusing on the statistical analysis of fire regimes *per se*. Notably, the chaparral, as a fire-prone ecosystem in Mediterranean climate regions, received the most attention during this decade. In the 2011−2020 period, the number of studies surged to 121. The papers focus even more on the intrinsic FRC and the influence of climate changes. A new critical area of inquiry emerged about the effects of fire suppression. Additionally, the frequent mention of fire ecology indicated a growing consolidation of research around a unified disciplinary theme. Since 2021, the 81 published studies demonstrate a continued rise of interest in FRC. A novel research hotspot focuses on fire as a disturbance to ecosystems and examines ecological and evolutionary responses at species and ecosystem levels. Meanwhile, the sustained attention to the long-term impacts of climate change on fire activity is evidenced by numerous studies referencing the Holocene epoch and charcoal as keywords. Human activities, particularly fire management practices, constitute the third major theme, highlighted by the high frequency of ‘fire management’ and ‘fire suppression’ in the literature.

### (c) Methodological approaches in fire regime change studies

The 233 FRC studies (apart from reviews and editorials) were broadly categorized into retrospective, predictive and observational studies. Retrospective analyses (64 studies) employ two distinct proxies across temporal scales: millennia-scale shifts are reconstructed using sedimentary records (charcoal, pollen and black carbon), and century-to-decadal changes rely on tree-ring data. Predictive studies (53 works) simulate future fire dynamics using climate scenarios paired with models such as dynamic vegetation models (e.g. LPJ, Orchidee), ecological niche models (e.g. MaxEnt) and process-based landscape frameworks (e.g. LANDIS). Observational assessments (116 estimates) focus on wildfires in recent decades and their impacts, generally integrate climate change, fire events and vegetation dynamics, leveraging field data and remote sensing (LiDAR, multispectral imagery) to track decadal-scale shifts in species distributions, population dynamics and ecosystem structure and functions.

The spatial scale (including extent, resolution or both) of FRC studies dictates methodological choices, especially in observational ones. At global and regional scales, climate-driven fire activity is analysed using remote sensing products, with MODIS data dominating large-scale studies over the past two decades. Regional- and landscape-scale analyses increasingly utilize Landsat and Sentinel-2 for finer spatio-temporal resolution, and visible infrared imaging radiometer suite (VIIRS) for night-time information. Landscape-scale studies also demand complex modelling (e.g. LANDIS) to integrate vegetation composition, fuel dynamics, environmental heterogeneity and human influences. These models are often validated through field surveys or experiments, particularly when examining ecological feedback, such as species adaptive traits or shifts in community composition.

Temporal scope defines data sources and analytical frameworks. Direct fire observations (ground records, remote sensing) are limited to approximately 50 years, while proxy-based reconstructions extend timelines: tree rings provide sub-millennial fire histories and sedimentary records resolve paleofire dynamics over millennia. Future projections rely on climate–fire relationship models (e.g. CMIP5/6 scenarios) to simulate shifts in fire probability, vegetation responses (e.g. net primary productivity (NPP), carbon emissions) and feedback loops. Bridging temporal and spatial gaps requires synthesizing disparate data—e.g. coupling sedimentary charcoal with modern remote sensing or validating model outputs against experimental field data—to unravel causal mechanisms and enhance predictive accuracy in an era of rapid environmental change.

## 3. Contribution of this thematic issue

This thematic issue comprises 17 papers, including 13 research articles, two reviews and two opinion pieces (table 2). Regarding the study areas covered in this special issue, five articles address FRC from a global perspective, four focus on North America, eight examine Eurasia, including an intercontinental comparison [25,26] and two analyse South America. The general studies incorporate relevant data from Africa or Australia, but no regional study was collected specifically for these two continents. Temporally, the majority of studies concentrate on FRC over recent decades; however, three studies provide a longer, more evolutionary perspective by investigating millennial- or centennial-scale historical FRC.

**Table 2.** The categorical information of the 17 papers in this special issue. Geography: NAm, North America; SAm, South America; WUI, wild–urban interface; theme: C, climate change; E, ecological impacts; F, fire and fire regime features; H, human activity; methodology: experi, experiments; field, field investigation; fossil, fossil and charcoal analyses, RS, remote sensing; sta, statistics.

authors	geography	ecosystems	theme	methodology
Charles *et al.*	general	general	F+E	review
Harrison *et al.*	general	general	F+C+H+E	RS+fossil+sta
Kamp *et al.*	Eurasia	temperate steppe	F+H+C	review
Li *et al.*	Asia	boreal & temperate forest	F+C+E	RS+sta
Little *et al.*	general	general	F+H+C	discussion
Little *et al.*	Europe	general	F+C	RS+model
Luo *et al.*	Asia	subtropic forests	F+E	field+experi
McGranahan & Wonkka	NAm	rangeland-WUI	F+H+E	RS+sta
Pausas *et al.*	NAm+Eurasia	boreal forest	F+C+E	RS+sta
Puig-Gironès *et al.*	general	general	F+H+E	discussion
Ramírez *et al.*	SAm	temperate deci-forest	F+E	field+sta
Segura-Garcia *et al.*	SAm	tropical savanna	F+C	RS+sta
Smith *et al.*	global	general	F+H	questionnaire
Tariyal *et al.*	Europe	boreal forest	F+H+C	tree-ring+sta
Wang *et al.*	NAm	boreal forest	F+C	RS+model
Wei *et al.*	Asia	boreal+subtropic forests	F+	isotope+experi

As to the sampling distribution of the collected papers, six studies explore FRC questions across ecosystem types. Specifically, boreal forests are a strong focus (six articles) due to their escalating wildfire activity and carbon emissions. Two studies address temperate and subtropical forests respectively, both incorporating boreal forest comparisons [27,28]. Grassland systems are represented by two articles: North American rangelands [29] and Eurasian temperate steppes [25]. Only one study specifically examines tropical savannas [30], with no Mediterranean vegetation-focused FRC research included.

The important role of remote sensing in FRC data acquisition is reflected in these studies, with at least eight of 13 employing satellite-derived spatial patterns of fire regime features. Field-based fire event sampling provides fine-scale vegetation–fire interaction data in only two studies. Long-term FRC reconstructions utilize charcoal deposits [31], tree-ring fire scars [32] and stable isotopes [28]. Additional methods include expert questionnaires [33] and atmospheric modelling indicators [34], while Wang *et al*. investigate climate drivers of fire behaviour through meteorological data [35]. Spatial substitution for temporal analysis emerges as a compromise strategy to address data resolution mismatches. Among 13 regional case studies, 10 emphasize spatial FRC patterns versus four temporal analyses. Spatio-temporal analytical frameworks predominantly employ cartographic, statistical and machine learning approaches.

The thematic composition of this collection demonstrates increasing integration in FRC research (figure 3). Ten and seven studies, respectively, examine climate change and anthropogenic drivers or feedback of FRC, with four addressing their interactions. Nine articles analyse FRC impacts on vegetation/species diversity, four of which concurrently consider climate change impacts and three anthropogenic influences. Smith *et al*. [33] pioneer a global synthesis of human fire-use strategies. Three specialized studies investigate plant community responses [36], animal–plant relationships [37] and plant trait adaptations [38]. Pausas *et al*. [26] make an inter-continental comparison of the top-down and bottom-up mechanisms of FRC in boreal forests. Harrison *et al*. [31] quantify the contribution of the comprehensive climate–human–vegetation interactions to FRC. Reviews and perspectives advance theoretical frameworks, including fire regime trait conceptualization [39], ecosystem-scale FRC effects [37], biodiversity implications [40] and complexities of anthropogenic impacts [25].

## 4. Patterns and drivers of fire regime changes and interaction with ecosystems

Drawing on the extensive body of literature on FRC from the past three decades, as well as the research compiled in this special issue, the following sections review the key characteristics of changing fire regimes, their primary natural and anthropogenic drivers and feedback mechanisms, and the resulting ecological impacts.

### (a) Changes in wildfire regimes

#### (i) Fire frequency and intensity change

Many regions are experiencing more frequent and severe fires, exceeding the historical range of variability. For example, boreal forests are experiencing more frequent and severe fires due to climate-driven changes in fire weather, lightning ignitions, snow cover and fuel moisture [41]. Alternatively, some regions in the Brazilian cerrado are experiencing increased fire activity while others see declines, accompanied by intensive agriculture management [30].

#### (ii) Expansion of fire-prone areas

Increasing fires have been recorded in Arctic tundra and tropical rainforests, which were historically less fire-affected [42,43]. Novel fire activity is increasingly observed in historically less fire-prone regions, such as the Arctic due to climate change, in desert due to plant invasion and tropical rainforests due to human activities (e.g. deforestation) in addition to droughts [30,39].

#### (iii) Altered fire seasonality

Fire seasons are becoming longer, with fires occurring earlier in the spring and later in the autumn, particularly in temperate regions. For example, earlier snowmelt and longer snow-free periods are extending fire seasons in boreal forests [44]. In Europe, persistent positive anomalies (PPAs) are associated with extreme fire weather, leading to increased burned areas during heatwaves [34].

### (b) Global hotspots of fire regime changes

— *Sub-Saharan Africa*: The impact of human activities and climate change has significantly decreased the fire regimes in savannas of sub-Saharan Africa, including fire suppression measures in protected areas [45,46].
— *Amazon and South America*: The interaction between deforestation and precipitation dynamics is changing fire regimes in the Amazon and other South American ecosystems [47,48]. In the Brazilian Cerrado, FRC are polarized, with increased fire activity in the north and declining activity in the south [30].
— *Arctic and boreal regions*: The significant warming in high-latitude regions of the Northern Hemisphere is driving earlier and prolonged fire seasons and increasing lightning activity. This intensifies extreme wildfires in boreal forests and introduces wildfires into the Arctic tundra, where fires were historically rare, and accelerates carbon emissions from forests and peatlands [49,50].
— *Asian subtropical semi-humid forests and savanna*: Research in Asia examines the role of climate change and human activity in shaping fire regimes in regions like China and India [51,52]. Fire frequency favours flammable plant species, creating a positive feedback loop between fire and vegetation [38].
— *Australia*: Mainly driven by extreme climate, the tropical savanna and Mediterranean shrublands in Australia are now a significant focus of FRC [53,54] with remarkable impacts on vegetation and biodiversity.
— *Mediterranean Basin and northern Europe*: Studies such as Pausas & Fernández-Muñoz [55] and Salis *et al.* [56] analyse FRC in Mediterranean ecosystems and the impact of historical land-use changes. PPAs are driving extreme fire weather and increasing burned areas, particularly in northern Europe [34].
— *Temperate North America*: In addition to boreal forests, the Mediterranean chaparral and temperate conifer forests have also experienced increasing extreme fires in the last decade [57,58]. Even in places such as the Great Basin Desert, the dispersal of invasive grasses has facilitated the spread of wildfires [21,59].

### (c) Dominant drivers of fire regime changes

#### (i) Climate change drivers

Wildfires typically occur in areas of sufficiently abundant and dry fuels and spread as the spatial continuity of combustibles increases. Climate change is often a dominant driver of changes in biomass combustibility, quantity and spatial distribution.

#### Climate warming trends and anomalies

Global warming is a widely observed phenomenon, with the rate of temperature increase displaying significant spatial heterogeneity, particularly a pronounced latitudinal gradient. Numerous studies have documented a marked rise in wildfire frequency in high-latitude regions over recent decades, especially an increase in high-intensity megafires across the Northern Hemisphere. This trend is closely linked to accelerated warming in these areas [39].

On one hand, prolonged warming alleviates low-temperature constraints on plant growth in high-latitude regions, enhancing vegetation productivity and annual litterfall. On the other hand, increased evapotranspiration under warming intensifies climatic aridity in areas without corresponding precipitation increases, elevating the flammability of both live and dead biomass. Warming trends are often characterized by more frequent and extreme heatwaves, which have been strongly associated with wildfire outbreaks [34].

The rate of warming varies across temporal and spatial scales, interacting with factors such as precipitation patterns, snowmelt timing and permafrost dynamics, and thus alters regional drought regimes. These changes influence the annual production, distribution and flammability of vegetation—particularly live and dead fine fuel—thereby modulating interannual variability in wildfire frequency. Such interactions underscore the complex mechanisms through which climate warming reshapes fire regimes, amplifying risks in vulnerable ecosystems.

This interplay between warming-driven environmental shifts and wildfire dynamics highlights the need for integrated models to predict future fire behaviour and inform adaptive management strategies in high-latitude regions.

#### Altered precipitation patterns and droughts

Global climate change is not only related to warming but also to regional shifts in precipitation patterns. Some regions exhibit declining rainfall, while others experience intensified climatic aridity due to warming or temporal concentration of rainfall. Especially in semi-humid and semi-arid zones, altered precipitation regimes—characterized by heavier rainstorms interspersed with prolonged dry spells—have become increasingly pronounced [60]. This pattern not only amplifies soil erosion and flood-induced vegetation damage, generating more dead fuels, but also exposes ecosystems to extended drought periods, heightening fire risks. In high-latitude regions, the rapid climate warming diminishes winter snowpack and accelerates spring snowmelt [61]. These changes enhance fuel availability, prolong the fire season and escalate the frequency and severity of wildfires. Both intra-annual and interannual climatic droughts have shown strong correlations with wildfire occurrence and intensity. Recent megafires in California, Australia and Portugal, for instance, coincided with significant regional drying trends [1,62]. Notably, large fires can further exacerbate local-scale droughts through feedback mechanisms, creating a self-reinforcing cycle of aridity and fire [63,64].

In humid and semi-humid forests, it is known that below-average precipitation elevates vegetation flammability, leading to increased fire activity. Conversely, in semi-arid tropical savannas, above-average rainfall boosts fuel production, paradoxically raising wildfire frequency during subsequent dry periods [65]. These divergent responses underscore the complex interplay between precipitation variability, fuel dynamics and fire regimes across ecosystems. Such findings highlight the need for adaptive land management strategies tailored to regional climatic shifts, ensuring resilience against escalating wildfire threats in a warming world.

#### Lightning occurrence change

Lightning is the driver of most naturally ignited wildfires, and accounts for a majority share of total ignitions in boreal and extratropical forests. Globally, lightning ignitions account for 77% of burned areas in extratropical intact forests [66]. Lightning-driven fires are associated with larger burned areas and higher severity, especially in remote regions with dense fuels [44,66]. Regionally, lightning explains >55% of interannual burned area variability in boreal forests [67]. In the US, lightning-ignited fires are 2.4 times more intense and 9.2 times larger than human-caused fires, dominating regions like the western US [44]. In the southeastern US, dry seasons have lengthened by 156 days over 120 years, increasing lightning fire potential [68]. In northeast China, the lightning fire season has been extended by 51 days since the late 1990s due to fuel drying, modulated by the Atlantic Multidecadal Oscillation (AMO) [41,69]. Lightning fires contribute disproportionately to burned areas in Portugal during extreme events [70].

Projections indicate an 11−31% increase in lightning strikes per degree of warming [66]. Warmer temperatures enhance convective potential and precipitation, driving lightning proliferation [43]. Therefore, lightning activity is expected to increase in the Arctic, boreal zones and northern midlatitudes but decline in the tropics [71].

#### Climate teleconnection dynamics

Sea surface temperature (SST) anomalies, driven by climate oscillations, such as the El Niño-Southern Oscillation (ENSO), AMO and Pacific Decadal Oscillation (PDO), significantly influence wildland fire activity by altering regional weather patterns, as a teleconnection mechanism. In the tropics and subtropics, ENSO strongly modulates drought intensity and fire weather conditions. For example, ENSO-driven droughts amplified understorey fires in the Amazon by 13 times compared with non-ENSO years [72]. Similarly, El Niño phases correlate with prolonged dry spells and heightened fire weather indices in Southeast Asia, particularly in peatlands [73]. The AMO and PDO further interact with ENSO to shape fire regimes in the middle and high latitudes; for example, warm AMO phases enhance lightning-ignited fires in boreal forests by promoting hot, dry conditions [41], while combined ENSO-PDO phases dictate fire risk in the Rocky Mountains [74].

Regional fire responses to SST anomalies vary due to differences in climatic and ecological contexts. In Canada, winter SST anomalies in the Atlantic and Pacific Oceans influence summer fire severity through lagged effects, with AMO-driven atmospheric patterns extending fire seasons [75,76]. In Australia, ENSO phases regulate fire frequency and burned area, with El Niño exacerbating drought and La Niña mitigating risks [77,78]. Conversely, in semi-arid regions of Mexico and Chile, ENSO-associated vapour pressure deficits (VPD) and heatwaves elevate fire probability [79,80]. Zhao *et al.* [81] found eight Northern Hemispheric teleconnections significantly affect 63% ± 2% of Arctic-boreal burned area [81], showing stronger impacts than that of ENSO. In northeast China, snowpack reduced and snow melt occurred earlier due to SST-linked warming, prolonging fire seasons [82]. These regional disparities highlight the role of SST anomalies in modifying localized fire weather drivers, including temperature, humidity and fuel availability.

#### (ii) Human activity drivers

The use of fire for warmth, repelling wild animals and obtaining food is regarded as one of the hallmarks of human civilization [83]. Fossil records including charcoal, pollen and other proxies reveal that worldwide wildfires and associated vegetation changes have been influenced to varying degrees by human activities. Humans have employed fire for thousands of years to alter vegetation and create land for cultivation or grazing [33]. Currently, human activities driving FRC across different ecosystems primarily include the following factors.

#### Human ignitions dominate most wildfire events

Human activities have increased the number of fire ignitions, particularly in regions where natural ignitions (e.g. lightning) are rare [84]. Over 50% area of ice-free land on Earth possesses traditional human ignition for various living purposes, particularly in rural and tribal areas [33]. In China, human-initiated wildfire ignitions comprise approximately 99% of wildfire events [85], especially concentrated on the traditional Chinese holidays [86]. Catastrophic mega-fires are mostly human-ignited in extreme fire weather and these fires result in more economic, human life and ecological damages [87].

#### Tropical deforestation increases fires

Tropical rainforests and seasonal forests, being the most productive and biodiverse biomes on Earth, are also highly sensitive to fire. In recent decades, deforestation and agricultural expansion have intensified the fragmentation of tropical rainforest landscapes. This repeatedly reported fragmentation in the pan-tropics [88], and specifically in Central Africa [89] and the Amazon [90], has reduced climatic humidity by increasing wind velocity, understorey light exposure and VPD—all of which enhance the flammability of understorey vegetation and litters. Thus, the rise in human-induced ignition sources combined with the increasing continuity of vertical forest structures has created favourable conditions for both the initiation and propagation of wildfires [91].

#### Expansion of agriculture in savanna regions depresses fires

An overview of the FRC studies in the Southern Hemisphere indicates a primary focus on tropical savannas (figure 2). Indeed, tropical and subtropical savanna comprise the largest percentage of global burned area [92], and this biome is undergoing significant decreases in fire frequency and fire carbon emissions due to a continuous expansion and intensification of agriculture in the last two decades [7,8].

#### The wild–urban interface as a front of fire regime changes

The global wildland–urban interface (WUI) has expanded significantly, with a 24% increase from 2001 to 2020. Despite the overall decrease in total global fire counts and burned area, the proportion of fires and burned areas within WUI zones rose by 23 and 35%, respectively, during this period [93]. Projections indicate continued WUI expansion, leading to heightened fire risks under most climate scenarios. Regions like the US, Canada, Australia, Southern Europe and China have experienced devastating WUI fires [29,93], driven by urbanization into fire-prone areas and exacerbated by extreme weather events. While WUI fires tend to be smaller in size compared with wildland fires, their proximity to communities amplifies their societal impact, including displacement, economic costs (e.g. $9 billion in Canada, 2016) and fatalities [94]. WUI fires are characterized by their complexity, involving human behaviour, infrastructure and their interactions with weather, topography and vegetation [94], with ignition hotspots often clustering near urbanized areas. The rapid urbanization process has driven the WUI area expansion, accompanied by land-use changes (artificial versus natural areas) and ignition sources (e.g. negligence, arson). The lack of robust building codes, retrofitting strategies and fire mitigation policies further exacerbates vulnerabilities.

#### Fire suppression policy accumulates fire risk with altered fire regimes

For over a century, the US and Canada have used fire suppression policies to limit the build-up of flammable vegetation and reduce human activities that accidentally or intentionally start wildfires in natural areas [95]. This policy effectively reduced wildfire frequency and burned area in regions with fire-prone vegetation, like mixed conifer forests and savanna [96], or in regions with dense population [97,98]. Fire suppression is expected to result in the subsequent development of dense forests with a build-up of hazardous fuels, and to transform the fire regime in areas of frequent-and-small fires to infrequent but destructive megafires [99]. Despite the widespread applications of fire suppression policy, the long-term monitoring of fire suppression impacts on fire regime is still rare. Therefore, the results are still undetermined and would present challenges for current and future fire management policy [99,100].

### (d) Ecological impacts of fire regime changes and ecosystem feedback

The changes in fire regimes driven by climate change or human activities both have complicated impacts on ecosystems, affecting vegetation, wildlife and ecosystem stability [100,101].

#### (i) Community compositional change and biodiversity loss

Increasing fire frequency in the Brazilian cerrado [30] and boreal forestsis leading to shifts in species composition, favouring fire-adapted species over fire-sensitive ones. In subtropical semi-humid forests, fire frequency favours flammable plant species, creating a positive feedback loop between fire and vegetation [38]. In the temperate Andes, fire-driven feedbacks are stabilizing pyrophilic shrublands and pyrophobic forests as alternative vegetation states [36]. Higher fire frequency generally puts fire-sensitive species, particularly those with long regeneration cycles, at risk of local extinction.

#### (ii) Carbon cycle disruption and modification

Increased fire activity in boreal forests releases large amounts of stored carbon into the atmosphere and converts these ecosystems from carbon sinks to sources, exacerbating climate warming and creating a positive feedback loop. Enlarging fire size also amplifies warming and carbon sources by triggering permafrost degradation and snowmelt, which releases stored carbon into the atmosphere and amplifies warming even further [62].

#### (iii) Soil and water degradation

Numerous local and global scale studies have demonstrated significant impacts of fires on soil degradation and water pollution. For example, intense fires in the cerrado and boreal forests are degrading soil quality and reducing water retention, impacting ecosystem productivity [30]. Intensifying fire regimes often outpace the adaptive capacity of native species and result in habitat degradation or even collapse [63].

#### (iv) Habitat fragmentation and bioinvasion

FRC can fragment habitats, reducing connectivity and resilience of ecosystems to future disturbances. Meanwhile, post-fire habitats are often favoured by alien species and provide a critical opportunity for the spread of invasive species, causing cascading stresses and disturbance on native species [102,103]. Such feedbacks can further alter fire regimes and ecosystem dynamics.

#### (v) Halted but accumulating fire risk

Fire suppression as a widely applied fire prevention strategy in North America and Europe since the 1970s [104,105], and since the 1990s in China, altered the natural fire regime, keeping vegetation succession away from fire disturbance and favouring fire-sensitive species. However, since it cannot hold the trend of broad-scale climate change, the climate-induced fire risk is actually accumulating along with vegetation biomass accumulation. It is worth noting that ecosystem characteristics, such as vegetation biomass and production, moisture, vertical and horizontal distribution, play a crucial role in the occurrence and spread of fire. As a product of specific fire regime, vegetation characteristics and species traits not only adapt to fire disturbance and habitat stress but also provide feedback on the fire process, making them one of the determining factors in the evolution of fire systems.

## 5. Summary and future prospects

Wildfire regimes are undergoing unprecedented changes at a global scale, with an overall trend of strengthening and expanding impact under the influence of anthropogenic climate change and human activities. There are still many knowledge gaps regarding the spatio-temporal characteristics of fire regimes in different regions, the driving mechanisms and ecological impacts of FRC. By reviewing the relevant literature of the last 30 years and summarizing the papers in this special issue, we see a trend in this field moving towards multidimensional and multiscale studies. The current hot topics include but are not limited to: mechanism analysis and comprehensive projections of FRC at multiple spatial and temporal scales; the scale-related responses and thresholds of ecosystem structure and functions to FRC; the contributions of vegetation status, climate changes and human activities to FRC in different ecosystems [26]; plant adaptation traits to fire regime across levels from gene to ecosystem; the causes and ecological impacts of vegetation transitions between fire-prone and fire-sensitive systems [39], including the transition between carbon sources and sinks; dynamics of vegetation restoration and ecosystem service changes under different fire regimes; the impact and risks of fire suppression policies and fuel management strategies on future fire regimes; the integration of indigenous knowledge [33] and local motivations for fire use with fire science and ecosystem management.

As a sample of the ongoing development of fire regime studies, this thematic issue reflects a trend towards comprehensive studies combining climate change, human activities and vegetation in understanding their interactions in fire activities. Specifically, this collection was weighted more towards high- and mid-latitude regions, due to their more drastic changes, and comparisons at continental scales. Filling the global knowledge gap requires further accumulation of retrospective, observatory and predictive studies.

The advancement of wildfire observation technology is a key driver for the development of research in this field. Passive and active sources of remote sensing, based on satellites, aircraft, drones and terrestrial platforms, provide unprecedented accurate information for real-time fire monitoring; AI models based on big data provide a third efficient approach to simulate and predict fire regimes, apart from statistical and mechanistic models. The integration of multisource data and multiscale models in the future will provide more real-time, accurate and efficient intelligent services for fire risk prediction, early warning and adaptive management.

As an inherent process of the Earth system, wildfire is a unique form of material and energy exchange between ecosystems and their environment and an important component of interactions between humans and nature. The sustainability of the coexistence between humans, ecosystems and fire regimes relies on interdisciplinary communication from environmental, ecological, social and economic perspectives, as well as consensus among science, policy and the public.

## Data Availability

Supplementary material is available online [106].
